# Antiparkinsonian drugs as potent contributors to nocturnal sleep in patients with Parkinson’s disease

**DOI:** 10.1371/journal.pone.0255274

**Published:** 2021-07-28

**Authors:** Soutarou Taguchi, Hirofumi Koide, Hiroko Oiwa, Miku Hayashi, Kazuhiro Ogawa, Chihiro Ito, Koji Nakashima, Tomoko Yuasa, Akihiro Yasumoto, Hiroaki Ando, Akifumi Fujikake, Takaaki Fukuoka, Keisuke Tokui, Masayuki Izumi, Yuka Tsunoda, Yuichi Kawagashira, Yohei Okada, Jun-ichi Niwa, Manabu Doyu

**Affiliations:** 1 Parkinson’s Disease Advanced Therapy Center, Aichi Medical University Hospital, Nagakute, Japan; 2 Department of Neurology, Aichi Medical University, Nagakute, Japan; Karolinska Institutet, SWEDEN

## Abstract

**Objective:**

To clarify whether antiparkinsonian drugs contribute to nocturnal sleep disturbances in patients with Parkinson’s disease (PD).

**Background:**

Although the major antiparkinsonian drugs L-dopa and dopamine agonists (DAs) have been found to affect sleep, little is known about the effects of specific drugs on sleep in PD patients.

**Methods:**

The study participants consisted of 112 PD patients (median age 72.5 years [inter-quartile range: IQR 65–79]; mean disease duration 8.44 years [standard deviation: 7.33]; median Hoehn and Yahr stage 3 [IQR 2–3.75]) taking one of three types of non-ergot extended-release DAs (rotigotine 32; pramipexole 44; ropinirole 36) with or without L-dopa (median daily total dosage of antiparkinsonian drugs 525.5 mg [IQR 376.25–658] levodopa equivalent dose [LED]). Participants were assessed using the PD Sleep Scale-2 (PDSS-2).

**Results:**

For the whole PD patient cohort, the PDSS-2 sleep disturbance domain score and the scores for item 1 assessing sleep quality and item 8 assessing nocturia were positively correlated with daily total dosage of antiparkinsonian drugs and dosage of L-dopa, but not with the dosage of DAs. Sub-analysis according to DA treatment revealed that DA dosage was not correlated with item 1 or 8 score in any of the subgroups. The LED ratio of rotigotine to the total dosage of antiparkinsonian drugs was inversely correlated with the item 1 score.

**Conclusions:**

These data suggest that antiparkinsonian drugs, in particular L-dopa, adversely affect nocturnal sleep in PD patients, especially in terms of sleep quality and nocturia. Thus, adjusting both the total dosage of antiparkinsonian drugs and the dose-ratio of L-dopa might be key actions for alleviating poor sleep quality in patients with PD. Among DAs, we found a clear positive correlation between the dose-ratio of rotigotine and sleep quality. Thus, partial L-dopa replacement with rotigotine could improve sleep quality in patients with PD.

## 1 Introduction

Parkinson’s disease (PD) is a chronic progressive neurological condition characterized by motor symptoms including resting tremor, rigidity, bradykinesia, and postural instability, as well as non-motor symptoms such as sleep disturbance, constipation, and orthostatic hypotension [1]. Various etiologies have been proposed for sleep disturbances in PD patients, including motor/non-motor symptoms, psychiatric symptoms, pharmacological treatments, coexisting primary sleep disorders, and PD per se [2]. The major antiparkinsonian drug types, L-dopa and dopamine agonists (DAs), have been reported to affect sleep. In particular, therapy-related sleep disturbances have been found to occur more frequently in PD patients treated with L-dopa than in those receiving DAs [3]. In PD patients with sleep disturbances, rotigotine or ropinirole were associated with a subjective diminution in sleep disturbance compared with placebo [4, 5], while pergolide was objectively associated with deterioration in sleep efficiency and increased instances of awakening during the night [6]. However, little is known about how the pharmacokinetic and pharmacodynamic profiles of specific dopamine drugs affect sleep disturbances. In this study, we examined the effects of antiparkinsonian drugs on nocturnal sleep in patients with PD treated with various antiparkinsonian drugs. The patients were surveyed about their nocturnal sleep disturbances and the survey results were analyzed with a focus on the effects of two major drug types: L-dopa and DAs.

## 2 Materials and methods

### 2.1 Subjects

From March 2019 to February 2020, we consecutively enrolled 112 PD patients who were taking one of three types of non-ergot extended-release DAs, with or without L-dopa. All participants were registered as patients at the Aichi Medical University Hospital (see Table 1). PD diagnoses were made according to the clinical diagnostic criteria for PD from the Movement Disorder Society (clinically established or probable PD) [7]. The patients were divided into three subgroups according to the DA with which they were currently being treated (32 were using the rotigotine transdermal patch [Rotigotine subgroup], 44 were taking pramipexole [Pramipexole subgroup], and 36 were being treated with ropinirole [Ropinirole subgroup]). As shown in Table 1, the three subgroups did not significantly differ in terms of gender composition, disease duration, daily total dosage of antiparkinsonian drugs, daily dosage of L-dopa, or Levodopa Equivalent Dose (LED [8]) ratio of DA (i.e., the dosage of DA to the total dosage of antiparkinsonian drugs). The uses of anti-cholinergics, amantadine, hypnotics or antipsychotics, and medication for urinary dysfunction or dementia, any of which could be potential confounding factors affecting sleep, were comparable between the three groups. Patients in the rotigotine subgroup were older than those in the pramipexole subgroup, patients in the rotigotine subgroup had a lower Hoehn and Yahr (HY) stage [9] than the other subgroups, and patients in the ropinirole subgroup had a lower daily dosage of DA than those in the other subgroups.

**Table 1:** Characteristics of the 112 PD patients according to DA subgroup.

	All 112 PD patients	Rotigotine subgroup	Pramipexole subgroup	Ropinirole subgroup	p value
n (gender, men: women)	112 (54: 58)	32 (12: 20)	44 (24: 20)	36 (18: 18)	0.329 ***
Age (years)	Median 72.5 [IQR 65, 79]	Median 76 [IQR 70, 81]	Median 69.5 [IQR 64, 76.75]	Median 70.5 [IQR 62, 78.75]	0.030 **
Disease duration (years)	Mean 8.44 [SD 7.33]	Mean 10.16 [SD 10.34]	Mean 6.98 [SD 4.47]	Mean 8.69 [SD 6.76]	0.170 *
Daily total dosage of antiparkinsonian drugs (mg, LED)	Median 525.5 [IQR 376.25, 658]	Median 605 [IQR 454.12, 726.12]	Median 500 [IQR 381.25, 613.37]	Median 505 [IQR 278.75, 655]	0.081 **
Daily dosage of L-dopa (mg, LED)	Mean 320.98 [SD 141.68]	Mean 335.94 [SD 127.78]	Mean 298.86 [SD 125.98]	Median 325 [IQR 200, 487.5]	0.416 *
Daily dosage of DA (mg, LED)	Mean 147.81 [SD 97.12]	Mean 188.44 [SD 108.10]	Mean 157.39 [SD 95.29]	Mean 100.00 [SD 66.59]	p < 0.001 *
LED ratio of DA	Mean 0.31 [SD 0.22]	Mean 0.32 [SD 0.17]	Mean 0.34 [SD 0.22]	Mean 0.27 [SD 0.25]	0.400 *
HY stage	Median 3 [IQR 2, 3.75]	Median 3 [IQR 3, 4]	Median 2 [IQR 2, 3]	Median 3 [IQR 2, 3]	p < 0.001 **
Occurrence of nocturia (%)	88	84	89	89	0.818 ***
Usage ratio of anti-cholinergic, amantadine (%)	5/ 7	6/ 6	2/ 11	8/ 3	0.471/ 0.323 ***
Concomitant usage ratio of					
hypnotics or antipsychotics (%)	28	34	32	17	0.197 ***
medication for urinary dysfunction (%)	23	25	23	22	0.959 ***
medication for dementia (%)	9	13	7	8	0.686 ***

DA, Dopamine agonist; HY, Hoehn and Yahr; IQR, Inter-quartile range; LED, Levodopa equivalent dose; PD, Parkinson’s disease; SD, Standard deviation.

p-values were calculated using one-way analysis of variance (ANOVA; *), the Kruskal–Wallis test (**), and the chi-squared test (***).

### 2.2 Clinical evaluation

Nocturnal sleep disturbances in the PD patients were assessed using the Japanese version of the PD Sleep Scale-2 (PDSS-2) [10, 11]. The PDSS-2 is a 15-item questionnaire used to assess nocturnal symptoms experienced in the past week. The items are divided into a sleep disturbance domain (items 1, 2, 3, 8, and 14), nocturnal motor symptoms domain (items 4, 5, 6, 12, and 13), and nocturnal symptoms of PD domain (items 7, 9, 10, 11, and 15). The total score ranges from 0 to 60, with higher scores indicating more disturbances.

### 2.3 Statistical analysis

Statistical analyses were performed using JMP version 14.2.0 (SAS Institute Inc., NC, USA). Continuous variables are presented as medians with inter-quartile range (IQR) or means with standard deviation (SD) depending on the normality of their distribution, ordinal variables are presented as medians with IQR, and nominal variables are presented as percentages. The Shapiro–Wilk’s test was used to examine whether variables showed a normal distribution. A one-way analysis of variance (ANOVA) was used for between-subgroup comparisons of disease duration (years), drug dosage (mg, LED), and LED ratio of DA, which all showed a normal distribution. The nonparametric Kruskal–Wallis test was used for between-subgroup comparisons of age (years), HY stage, and drug dosage (mg, LED), which were not normally distributed. The chi-squared test was used for between-subgroup comparisons of gender composition, ratio of nocturnal urinary dysfunction, and concomitant usage ratio of medication that may affect sleep in PD. Spearman’s rank correlation coefficient was used to test for correlations between PDSS-2 (points), drug dosage (mg, LED), age (years), disease duration (years), and HY stage. If there was any difference in patient characteristics between the three subgroups, the Tukey-Kramer or Steel-Dwass adjustment was used to counteract the problem of multiple comparisons. Multiple regression analysis was used to determine the exposure/confounding factors. The level of significance was set at p < 0.05.

### 2.4 Ethical statement

This study was approved by the ethical review board of Aichi Medical University (No. 2019-H133) and conformed with the Declaration of Helsinki. All participants provided written informed consent.

## 3 Results

**Fig 1:**
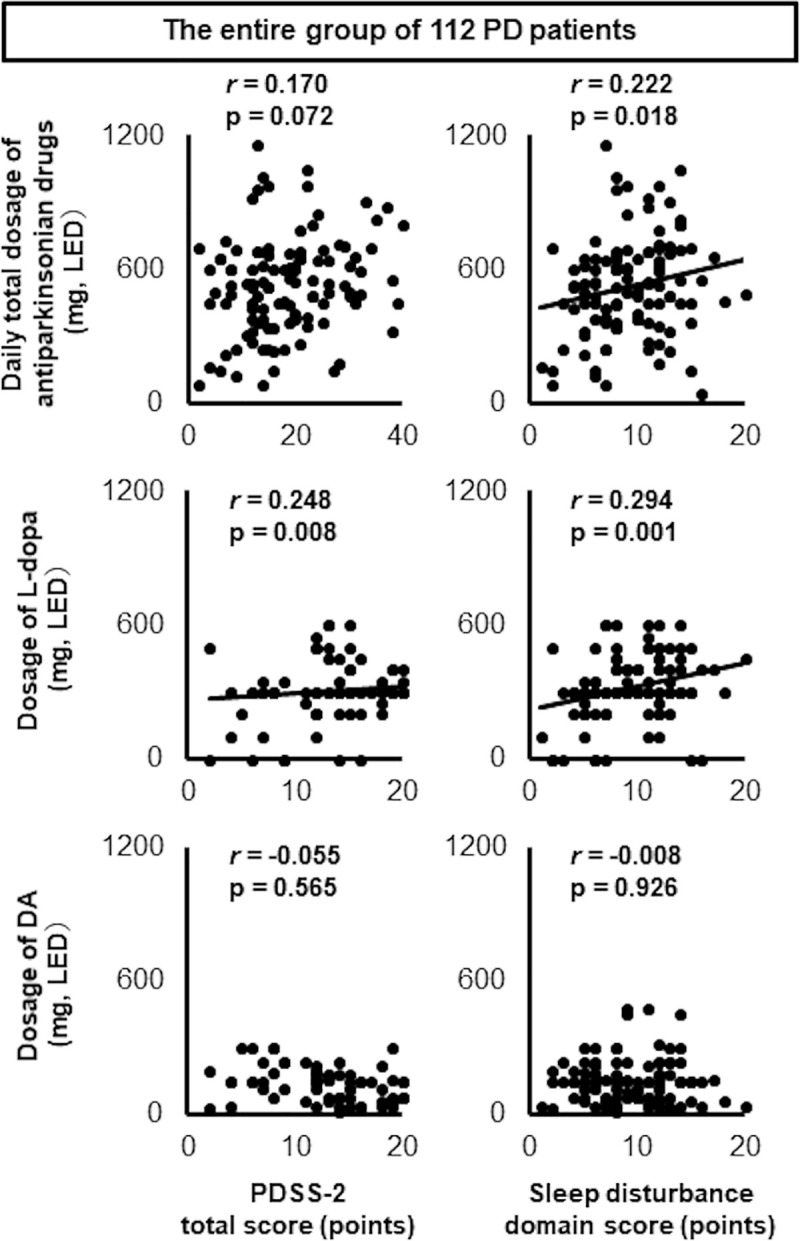
Scatter plots showing PDSS-2 total score and sleep disturbance domain score against drug dosage. The PDSS-2 total score was significantly correlated with the dosage of L-dopa, and the correlation coefficient was much higher than that between PDSS-2 and DAs. Among the three subdomains of the PDSS-2, the sleep disturbance domain score was significantly positively correlated with the daily total dosage of antiparkinsonian drugs and the dosage of L-dopa, with L-dopa showing higher correlation coefficients than DAs. The DA dosage was not significantly correlated with the total PDSS-2 score or the sleep disturbance domain score. P-values were calculated using Spearman’s rank correlation coefficient.

First, we analyzed the correlation between antiparkinsonian drug treatment and sleep in 112 PD patients. We found that the PDSS-2 total score was significantly positively correlated only with the dosage of L-dopa, and that this correlation coefficient was much higher than that between DAs and PDSS-2 total score. Among the three domains of the PDSS-2, only the score in the sleep disturbance domain was significantly positively correlated with the daily total dosage of antiparkinsonian drugs and the dosage of L-dopa, with the DA dosage not being significantly correlated with either the total PDSS-2 score or the score of any of the three domains. The sleep disturbance domain score showed a higher correlation with L-dopa dosage than with DA dosage, which suggests that in the studied cohort of PD patients, L-dopa had a greater effect on sleep disturbance than DAs (Fig 1 and S1 Fig).

**Fig 2:**
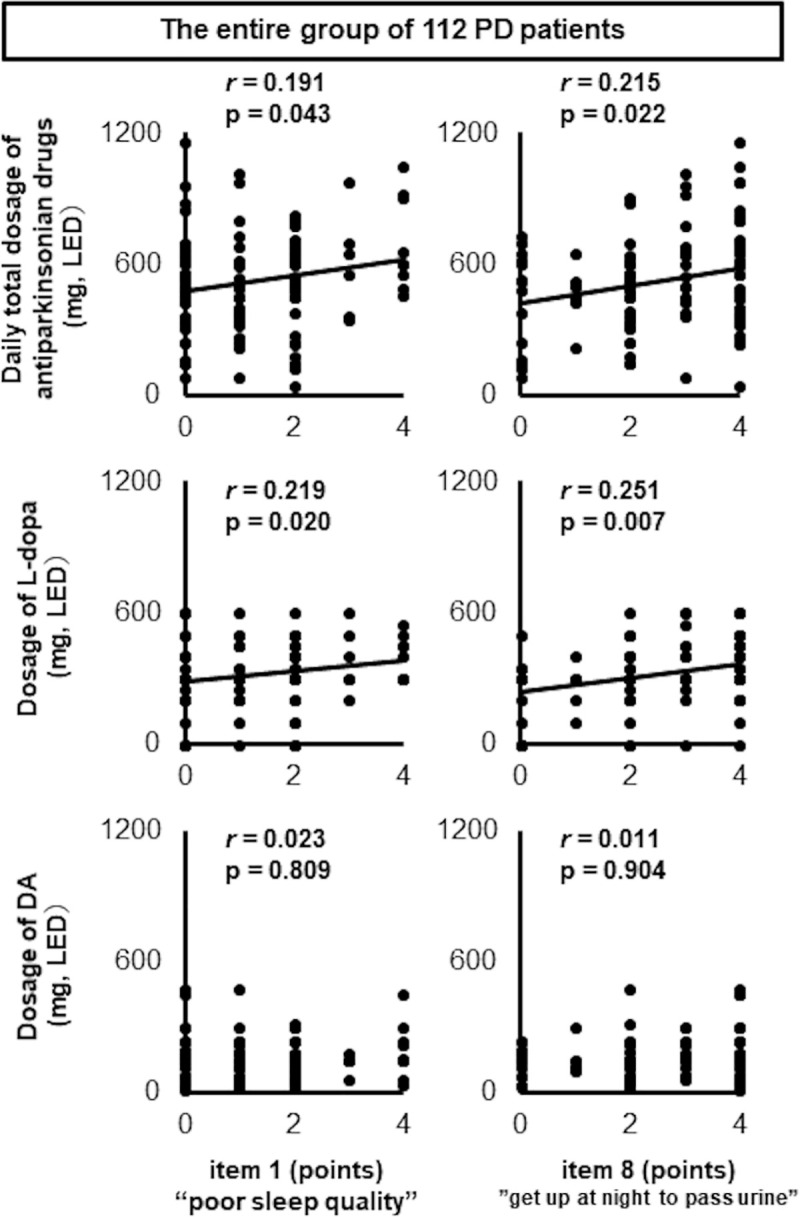
Scatter plots showing scores for items 1 and 8 in the PDSS-2 sleep disturbance domain against drug dosage. Scores on PDSS-2 items 1 and 8 were significantly correlated with the daily total dosage of antiparkinsonian drugs and the dosage of L-dopa. These correlation coefficients were higher than those with DAs. P-values were calculated using Spearman’s rank correlation coefficient.

Among the five items in the PDSS-2 sleep disturbance domain, item 1 assessing sleep quality and item 8 assessing the number of times patients get up at night to pass urine were both significantly correlated with the daily total dosage of antiparkinsonian drugs and the dosage of L-dopa. These correlation coefficients with L-dopa were higher than those with DAs, which suggests that L-dopa dosage had a greater effect on items 1 and 8 than did DA dosage. (Fig 2). This was not the case for the other items, which assessed difficulties with falling asleep, staying asleep, and feeling tired/sleepy after waking in the morning (items 2, 3, and 14, respectively; S2 Fig).

We then performed a multiple regression analysis to identify exposure/confounding factors and found that the item 1 score was significantly affected by the dosage of L-dopa, but not by disease duration, HY stage, or patient age, or adjustment for any of these three effects (Table 2). We also found that both L-dopa dosage and patient age adversely influenced the score for item 8, which measured the frequency of nocturnal urination, whereas disease duration and HY stage did not affect the score.

**Table 2:** Multiple regression analysis to identify predictors for the item 1 and 8 scores.

	Item 1				Item 8			
	B	SE	p value	95% CI	B	SE	p value	95% CI
Dosage of L-Dopa(mg)	0.200	0.0008	0.039	0.00008, 0.003	0.249	0.0008	0.005	0.0007, 0.004
Age (years)	-0.022	0.013	0.814	-0.029, 0.023	0.290	0.014	0.001	0.017, 0.073
Disease duration (years)	0.033	0.015	0.727	-0.025, 0.036	-0.086	0.017	0.346	-0.050, 0.017
HY stage	-0.021	0.134	0.829	-0.295, 0.237	-0.059	0.148	0.547	-0.383, 0.204

B, Unstandardized regression coefficient; CI, Confidence Interval; HY, Hoehn and Yahr; SE, Standard Error.

P-values were calculated using multiple regression analysis.

**Fig 3:**
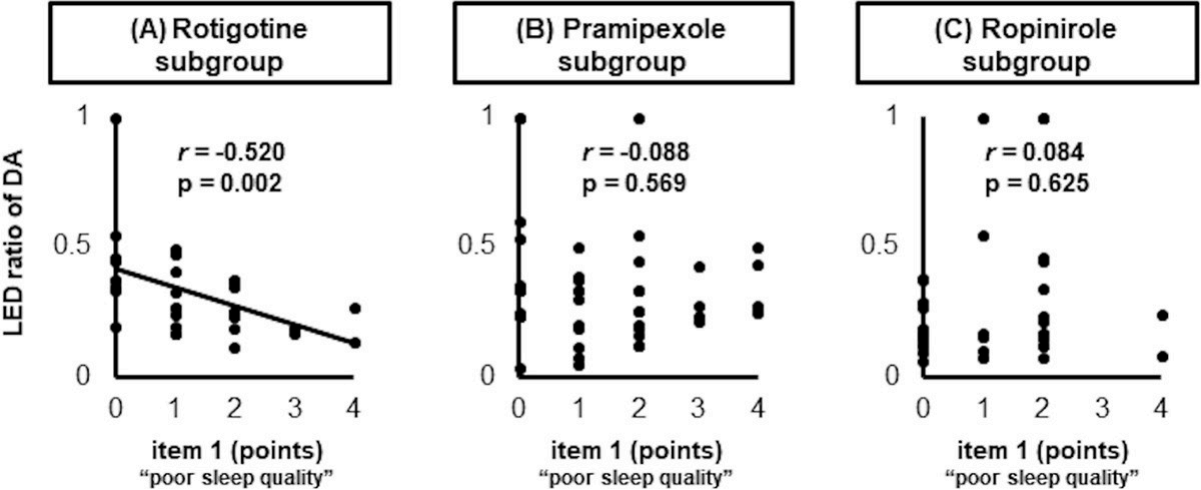
Scatter plots showing the item 1 score and ratio of DA. (A) The LED ratio of rotigotine was inversely correlated with the score for item 1. (B, C) Analyses of the pramipexole and ropinirole subgroups demonstrated no correlations between LED ratio and item 1. P-values were calculated using Spearman’s rank correlation coefficient.

Finally, we conducted a subgroup analysis according to the DAs received by each participant and correlations with the item 1 and 8 scores, to verify whether the three DAs differed in terms of their influence on sleep quality and nocturia. We found that the LED ratio of rotigotine to the total daily dosage of antiparkinsonian drugs was inversely correlated with the score for item 1 (Fig 3A), but not the actual rotigotine dosage (S3 Fig). However, the score for item 8 was not correlated with either the LED ratio or the rotigotine dosage (S3 and S4 Figs). Moreover, a significant correlation such as that demonstrated in the rotigotine subgroup, was not observed in the analyses of the pramipexole and ropinirole subgroups (Fig 3B and 3C, S3 and S4 Figs).

## 4 Discussion

In the present study, we retrospectively and cross-sectionally surveyed nocturnal sleep disturbances in patients with PD, investigating these in relation to antiparkinsonian drugs, with a focus on the effects of two major drug types: L-dopa and DAs. As shown in Fig 1, in the entire group of 112 PD patients, the PDSS-2 total score was significantly positively correlated only with L-dopa, and although this correlation coefficient was relatively small, it was higher than that between the PDSS-2 total score and DAs. This dose-dependent relationship between PDSS-2 score and L-dopa dosage is consistent with a previous report [12]. The correlation between L-dopa and the PDSS-2 sleep disturbance domain was sufficient to ensure that the daily total dosage of antiparkinsonian drugs also correlated with this domain, whereas in contrast, none of the DA types correlated with any of the three PDSS-2 domain scores. Furthermore, among the five items in the sleep disturbance domain, the scores for items 1 and 8 were significantly correlated with L-dopa dosage and the daily total dosage of antiparkinsonian drugs. These correlation coefficients were much higher than those with DAs. Thus, our data demonstrate that L-dopa was associated with deterioration in nocturnal sleep in PD patients via disturbances to nocturnal sleep quality and nocturia, whereas DAs did not show any significant associations with sleep.

The relationships between sleep disturbance and disease duration, severity, and age are unclear with respect to previous studies [12, 13]. To precisely assess the relationships between drug dosage and sleep quality or nocturia, we also examined the influence of disease duration, HY stage, and patient age. Multiple regression analysis showed that L-dopa adversely affected sleep quality in PD patients, even after adjusting for the effects of disease duration, severity (HY stage), and age, which did not have any significant effect on sleep quality. However, nocturia was adversely affected by both patient age and L-dopa dose, with aging showing a greater effect than L-dopa (unstandardized regression coefficient = 0.290 *vs*. 0.249), although nocturia was not affected by disease duration or severity (Table 2).

Our serial analyses indicate the possibility that L-dopa could latently disturb nocturnal sleep in PD patients. Therefore, we consider it important to carefully regulate the L-dopa dose. It was reported that the frequency of sleep disorders was lower in patients who received DAs than in those who received L-dopa (5.5% *vs*. 8.7%) [3]. Thus, the use of non-L-dopa drugs such as DAs as a substitute for L-dopa might be beneficial for avoiding or reducing L-dopa therapy-related sleep disturbances.

On the basis of our initial findings, we focused on the correlations between DA use and the scores for items 1 and 8 of the sleep disturbance domain of PDSS-2. Among the subgroup analyses on the three types of DAs, only the LED ratio of rotigotine was inversely correlated with the score for item 1 (Fig 3A). Although the patient characteristics of DA dosage, age, and HY stage differed among the three subgroups (Table 1), they did not affect sleep quality (Fig 2 and Table 2), and therefore did not alter the interpretation of the sub-analysis results. Therefore, we believe that these results indicate that only rotigotine had a beneficial effect on L-dopa therapy-related sleep disturbances. Therefore, replacing L-dopa with rotigotine might improve sleep quality and patient satisfaction. A previous study reported that D1 dopamine receptor stimulation favorably influenced sleep architecture [14], and rotigotine has a D1 receptor affinity that is comparable to that of L-dopa, unlike pramipexole and ropinirole [15]. Differences in pharmacodynamic profiles with respect to DA action at the D1 receptor may partially explain why only rotigotine substitution produced favorable outcomes for item 1 of the sleep disturbance domain. In addition, D1 receptor stimulation inhibits the micturition reflex [16], but rotigotine had no significant effect on item 8 (i.e., nocturia) in this study (S3 and S4 Figs). As we speculated above, aging could adversely affect nocturia to a greater degree than L-dopa. Thus, control of micturition via only the D1 pathway is not likely to have a substantial impact on nocturnal urination.

Unfortunately, the reason for L-dopa (a precursor of dopamine and a D1 receptor stimulant) decreasing sleep quality in patients with PD was not elucidated in the present study. However, it was reported that non-optimal dopamine concentrations in the brain may disturb parkinsonian sleep [17], and L-dopa-carbidopa continuous infusion gel therapy, which was found to produce longer and more-stable D1 stimulation, improved sleep quality in PD patients [18]. Therefore, considering that D1 receptors are substantially lower in the parkinsonian brain, it is reasonable to speculate that L-dopa could cause inappropriate excessive and phasic activation of D1 receptors, resulting in adverse effects on sleep quality. While the long-acting D1 agonist pergolide worsened sleep efficiency in patients with PD [6], it ameliorated restless legs syndrome [19], although the studies used actigraphy or polysomnography as an objective measure of nocturnal activity, which did not necessarily reflect patient subjectivity [20]. Therefore, we consider that partial L-dopa replacement with rotigotine, a non-phasic and continuous D1 agonist, could improve the subjective sleep status of patients with PD.

This study is subject to several limitations, although we consider the numbers of included patients to be appropriate as the correlation analyses showed significant p-values. The present exploratory study was a retrospective cross-sectional design, and we were not able to directly compare the effects of each drug nor consider comorbidities that may affect sleep. Further prospective studies are required to enable direct comparisons of the drug effects. Our present study focused on the effects of antiparkinsonian drugs on subjective sleep disturbance, which may not necessarily reflect objective sleep parameters [20]. Therefore, further studies using more objective parameters such as polysomnography may be beneficial for understanding associations between sleep disturbance and antiparkinsonian treatment in PD patients, although subjective parameters are also important in terms of evaluating the quality of life of patients with PD. Finally, in addition to dopamine, other neurotransmitters (e.g., serotonin and noradrenaline) are implicated in sleep disturbances in PD patients [2]. Future studies should consider the involvement of additional neurotransmitters in sleep disorders.

## 5 Conclusions

We were able to draw several conclusions from our data. L-dopa, the major antiparkinsonian drug, appears to adversely affect nocturnal sleep in PD patients, especially in terms of sleep quality and nocturia. Adjusting both the total dosage of antiparkinsonian drugs and the dose-ratio of L-dopa might be a key action in reducing or eliminating therapy-related sleep disturbances in patients with PD. Concerning DAs, we found a clear relationship between sleep quality and the dose-ratio of rotigotine; thus, partial L-dopa replacement with rotigotine could improve sleep quality and sleep satisfaction in patients with PD.

## Supporting information

S1 Fig Scatter plots showing the nocturnal motor symptoms, nocturnal symptoms domain scores, and drug dosage. (TIF)

S2 Fig Plots showing the scores for items 2, 3, and 14 of PDSS-2 against drug dosage. (TIF)

S3 Fig Plots showing the scores for items 1 and 8 of PDSS-2 against dosage of DA. (TIF)

S4 Fig Plots showing the scores for item 8 of PDSS-2 against the LED ratio of DA. (TIF)
