# Supplementary figures and images for: Antiparkinsonian drugs as potent contributors to nocturnal sleep in patients with Parkinson’s disease
Source: PLoS One. 2021 Jul 28;16(7):e0255274. doi: 10.1371/journal.pone.0255274 (PMC8318227; doi:10.1371/journal.pone.0255274)

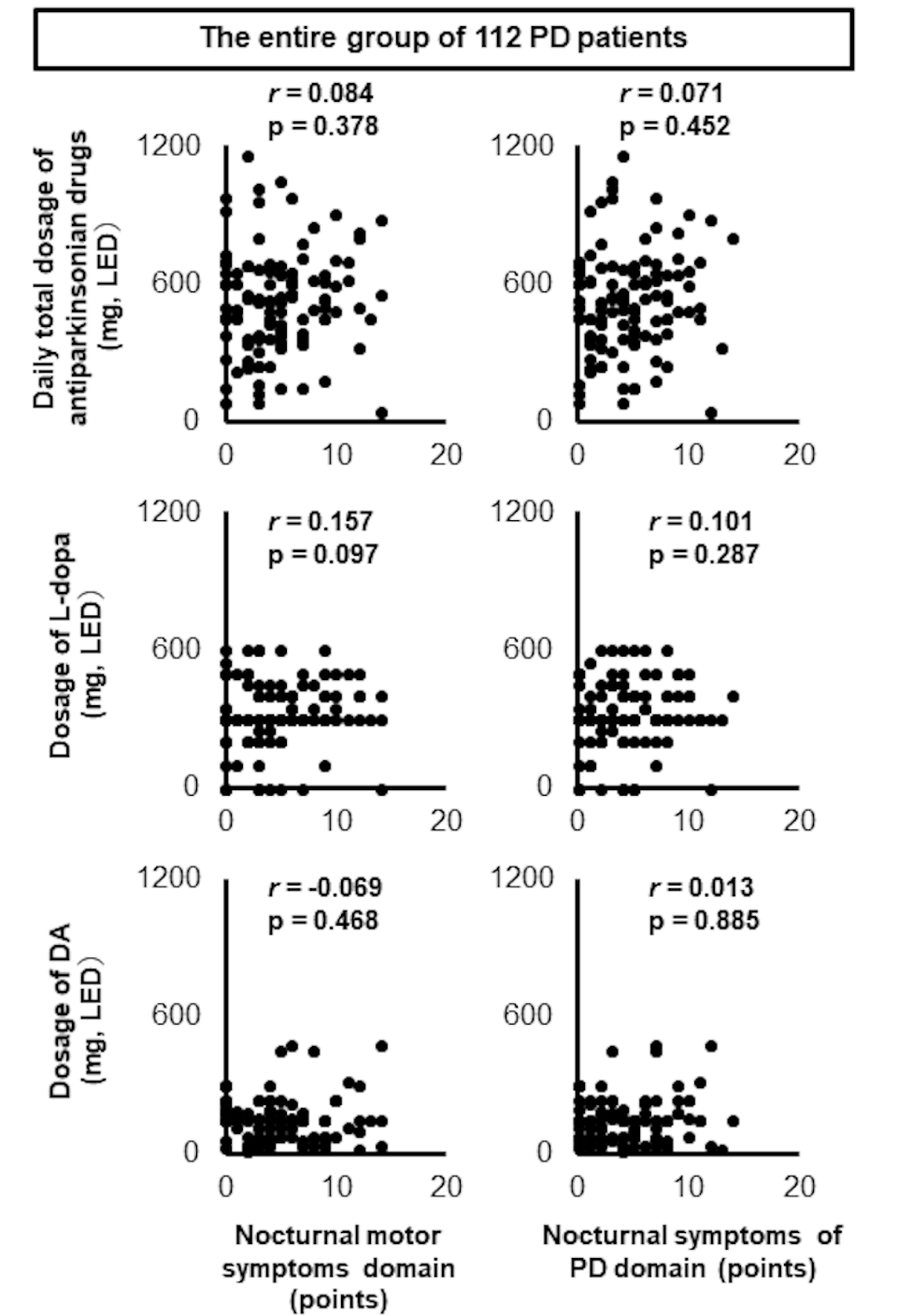

Supplement: S1 Fig — (TIF) [file pone.0255274.s001.TIF]

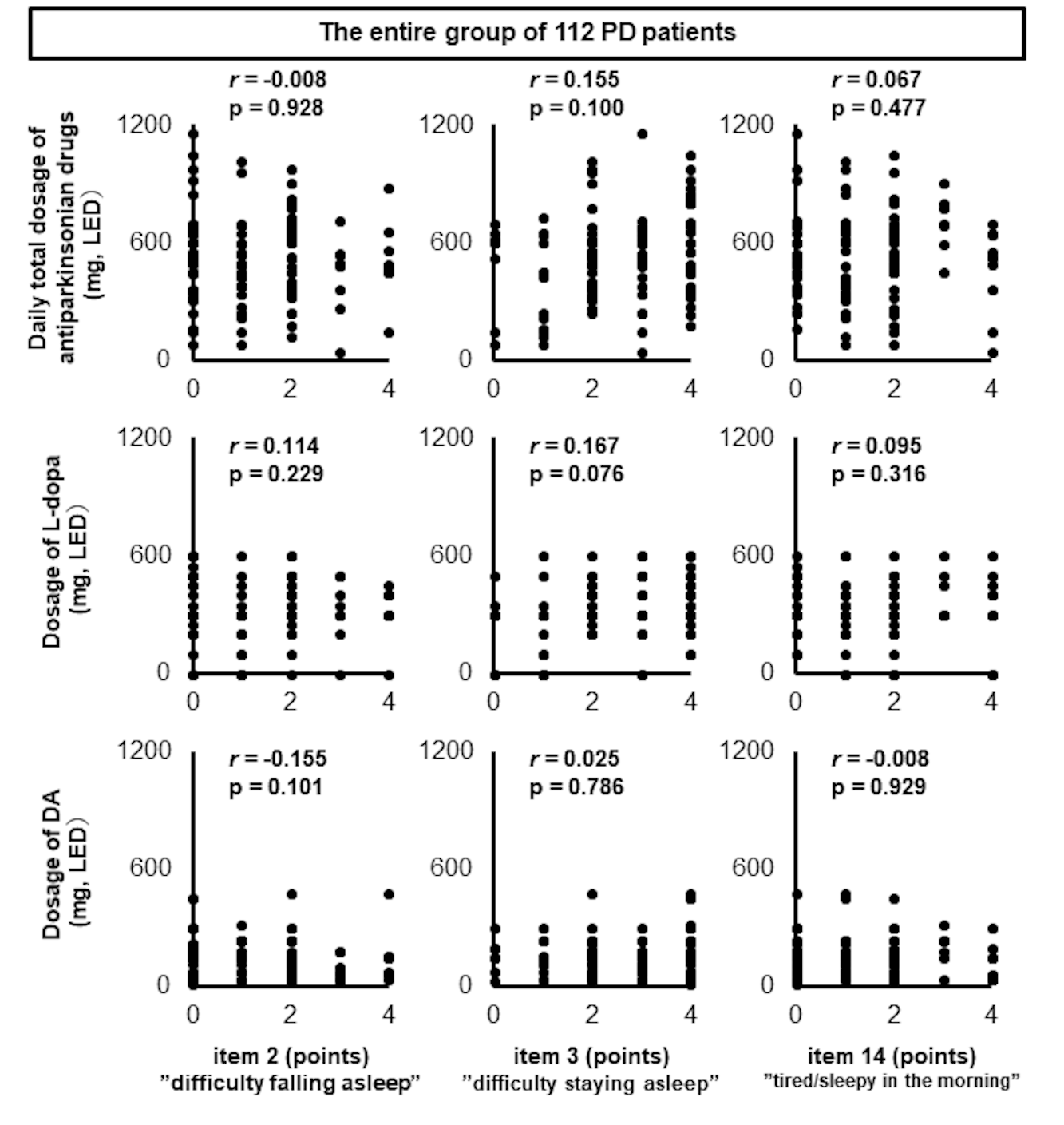

Supplement: S2 Fig — (TIF) [file pone.0255274.s002.TIF]

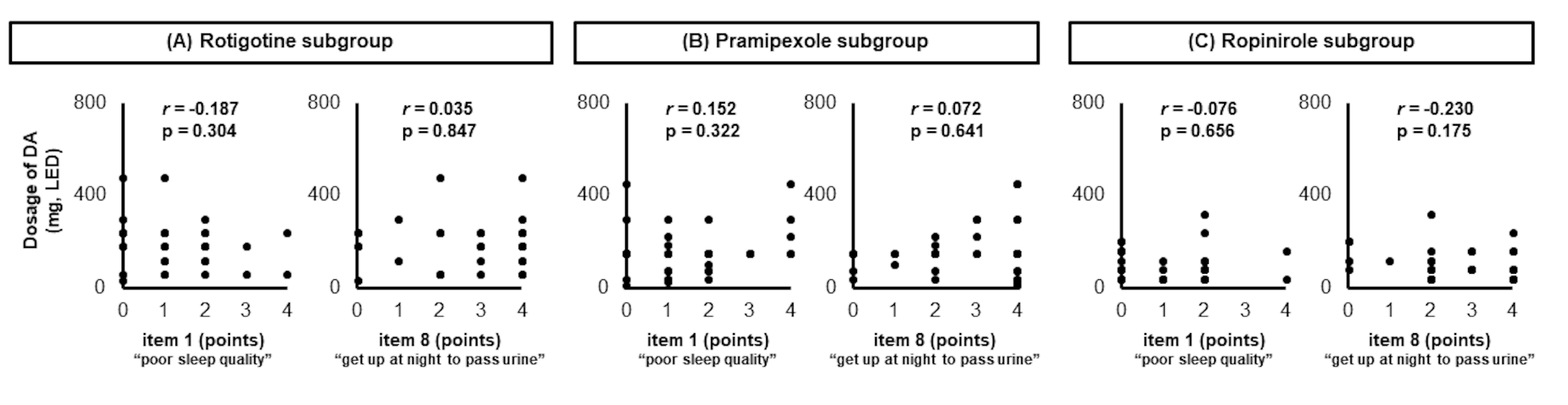

Supplement: S3 Fig — (TIF) [file pone.0255274.s003.TIF]

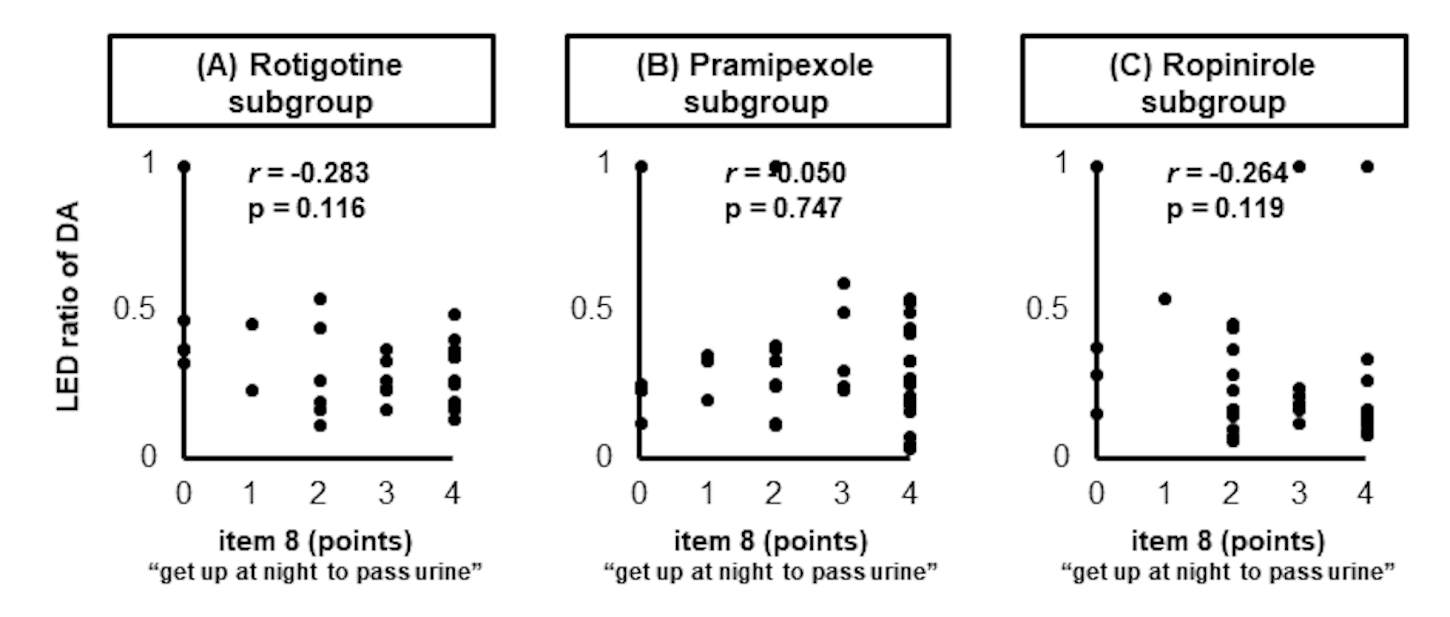

Supplement: S4 Fig — (TIF) [file pone.0255274.s004.TIF]
